# Clinical outcomes of lateral ear canal resection performed in routine clinical practice for chronic otitis externa in dogs: a retrospective descriptive study of 58 ears (2016‐2025)

**DOI:** 10.1111/jsap.70156

**Published:** 2026-06-25

**Authors:** K. Kiriki, A. Yabuzoe, K. Iyori

**Affiliations:** ^1^ Kiriki Animal Hospital Miyazaki Japan; ^2^ Yabuzoe Animal Hospital Wakayama Japan; ^3^ 1sec. Co., Ltd. Tokyo Japan

## Abstract

**Objectives:**

To retrospectively evaluate the long‐term clinical outcomes of lateral ear canal resection performed as part of routine clinical practice for chronic otitis externa in dogs, and to re‐evaluate the practical utility of lateral ear canal resection from the perspective of contemporary treatment goals emphasising long‐term maintenance of a manageable condition.

**Methods:**

A retrospective descriptive study was conducted involving 40 dogs (58 ears) that underwent lateral ear canal resection performed by a single non‐specialist surgeon at a single primary care facility between May 2016 and March 2025, with a minimum postoperative follow‐up of 12 months. Lateral ear canal resection was performed whenever chronic otitis externa was considered difficult to manage with medical treatment alone, without the application of predefined staging criteria. Changes in the annual frequency of ear cleaning before and after surgery were recorded, and owner assessments were conducted at or near the final follow‐up visit at least 12 months postoperatively.

**Results:**

Over a median follow‐up period of 952 days (interquartile range 589.5 to 1617), no case progressed to end‐stage ear canal disease or required conversion to total ear canal ablation with lateral bulla osteotomy. No serious postoperative complications were observed. The median annual ear‐cleaning frequency decreased markedly from 6.74 (interquartile range 2.06 to 14.30) before surgery to 1.58 (interquartile range 0.42 to 3.77) after surgery. The median postoperative pruritus score improved from 100% preoperatively to 20% (interquartile range 10 to 35). Owner assessment scores exceeded a mean of 3.89 across all items.

**Clinical Significance:**

Lateral ear canal resection performed in routine clinical practice before the development of overt end‐stage ear canal disease may suppress progression of chronic otitis externa and support long‐term disease manageability. The findings of this study suggest that lateral ear canal resection can be performed in a primary care setting without specialist qualifications or specialised equipment, and may contribute to avoiding progression to total ear canal ablation with lateral bulla osteotomy in similar clinical contexts. In contemporary otitis management, lateral ear canal resection warrants re‐evaluation not only as a salvage procedure but as a treatment option to be considered earlier in the disease course.

## INTRODUCTION

Chronic otitis externa is one of the most commonly encountered conditions in dogs and is recognised as a multifactorial disease involving a complex interaction of primary, secondary, predisposing and perpetuating (PSPP) factors (Jacobson, 2002; Nuttall, 2023). Many cases require long‐term management to prevent recurrence following remission, and it has increasingly been recognised that maintaining a manageable condition over the long term, rather than achieving complete cure, represents a realistic treatment goal in contemporary practice (Nuttall, 2023).

Lateral ear canal resection (LECR) is a surgical procedure involving resection of the lateral wall of the auricular cartilage to improve drainage and ventilation and has long been utilised as a minimally invasive surgical option to facilitate the management of chronic otitis externa while preserving hearing (Gregory & Vasseur, 1983; Tufvesson, 1955). Total ear canal ablation with lateral bulla osteotomy (TECA/LBO), by contrast, is well established as an effective definitive treatment for end‐stage chronic otitis externa, with clearly defined indications. LECR has historically been considered to have a lower success rate than TECA/LBO when complete cure is used as the primary outcome measure (Doyle et al., 2004; Gregory & Vasseur, 1983; Sylvestre, 1998), yet large‐scale reports evaluating the long‐term outcomes of LECR remain limited, and re‐evaluation from a contemporary perspective is warranted.

The objective of this study was to retrospectively evaluate the long‐term clinical outcomes of LECR performed as part of routine clinical practice at a single primary care facility, and to re‐evaluate the practical utility of LECR from the perspective of contemporary treatment goals emphasising long‐term maintenance of a manageable condition.

## MATERIALS AND METHODS

### Case selection

This study is a single‐centre retrospective descriptive study, and the findings should be interpreted as hypothesis‐generating. No formal inclusion or exclusion criteria for LECR were predefined at the time of clinical decision‐making. LECR was performed as part of routine clinical practice whenever chronic or recurrent otitis externa was considered difficult to manage with medical treatment alone and progression to ear canal stenosis requiring TECA/LBO was anticipated. For the purposes of this retrospective study, medical records were reviewed to identify cases in which LECR had been performed between May 2016 and March 2025 at a single primary care facility by a single non‐specialist surgeon, and for which perioperative and postoperative follow‐up data of at least 12 months were available. For cases that had died at the time of data collection, the date of the final visit prior to death was used as the final follow‐up date; for surviving cases, the date of the most recent visit was used. Retrospective review of the final medical records confirmed that all included cases met one or more of the following clinical situations: persistent failure to improve despite repeated medical treatment with ongoing concern regarding future progression of ear canal stenosis; persistent purulent discharge confirmed on otoscopic and physical examination that failed to resolve despite prolonged antimicrobial therapy guided by culture and sensitivity testing; failure to respond to multiple antimicrobial agents in cases where culture and sensitivity testing was not feasible; or difficulty performing ear cleaning without sedation due to behavioural factors in cases of persistent chronic otitis externa where progression of stenosis was anticipated without regular ear cleaning.

#### Exclusion criteria

Cases in which severe horizontal ear canal stenosis was confirmed on otoscopic examination and LECR was considered unlikely to be beneficial, or in which marked horizontal ear canal stenosis was identified intraoperatively, were managed with TECA/LBO or advised to undergo TECA/LBO instead of LECR. No cases were identified in which TECA/LBO had been recommended, but LECR was selected at the owner's request.

### Medical management

All dogs presenting with clinical signs of otitis externa underwent a history and general physical examination, followed by otoscopic evaluation of the external ear canal. When cerumen or purulent discharge was identified, smears were stained and examined microscopically for bacteria, fungi and cells to guide therapeutic selection. Ear cleaning was performed in the majority of cases presenting with otitis externa. When indicated, a ceruminolytic agent was instilled into the ear canal for approximately 10 minutes prior to flushing with chlorhexidine gluconate solution, followed by gentle manual massage. Ear cleaning was repeated at approximately weekly intervals according to the condition of the ear canal and continued until clinical remission was achieved. In cases where tenacious cerumen or debris was identified in the vicinity of the tympanic membrane on otoscopic examination, gentle flushing was performed using a feeding catheter. Ear cleaning was performed without anaesthesia in most cases, except where the dog was uncooperative or showed overt signs of pain, in which case anaesthesia was occasionally employed.

With regard to medications, a topical corticosteroid preparation was administered following ear cleaning in the majority of cases. Topical antifungal agents were used when Malassezia organisms were identified on cytological examination in association with pruritus. For suppurative otitis externa, ear cleaning was prioritised and empirical antimicrobial use was avoided unless the condition was severe. When improvement was not achieved, systemic antimicrobial therapy was administered based on culture and sensitivity results; in cases where culture and sensitivity testing was not feasible, amoxicillin, cefalexin or enrofloxacin were commonly selected. In cases with marked pruritus or pain, a single subcutaneous injection of prednisolone or a short course of oral prednisolone for approximately 1 week was administered. Oclacitinib was commonly used for pruritus management in non‐severe cases.

With regard to dietary management, a detailed dietary history was obtained when food allergy was suspected based on the clinical history and when the response to standard medical treatment was poor, or when requested by the owner. When dietary therapy was deemed appropriate and owner consent was obtained, a novel protein diet based on the dietary history was introduced. If the response was insufficient, multiple novel protein diets or hydrolysed protein diets were trialled sequentially as an elimination diet, and the most effective diet was continued. Food challenge testing was performed when requested by the owner to identify tolerable ingredients.

In cases of erythematous or ceruminous otitis externa where the response to routine ear cleaning and topical corticosteroids was poor and excessive cerumen accumulation or persistent clinical signs were observed, the topical preparation was changed to a combination product containing fluocinolone acetonide, polymyxin B, miconazole and triamcinolone following ear cleaning. This preparation was administered at intervals of 7 to 10 days, with two to three administrations constituting one course of treatment. If the response remained poor, the preparation was switched back to a single‐agent topical corticosteroid with consideration of the risk of antimicrobial resistance, and the ear‐cleaning interval was shortened to 4 to 5 days to achieve intensive cleaning. Oral antifungal therapy was added when Malassezia was identified on cytological examination of ear debris. In cases where improvement remained insufficient, a switch to a combination preparation containing florfenicol and trimethoprim‐sulfadiazine was attempted. When improvement was still not achieved, or when three or more courses of the aforementioned combination preparation were required within a six‐month period, medical management at this facility was considered insufficient. Video otoscopy was not utilised in any case included in this study.

### Surgical procedure

The surgical procedure was performed in accordance with previously reported methods, involving resection of the lateral wall of the auricular cartilage to improve drainage and ventilation (Fossum, 2019). Anaesthesia was induced and maintained with atropine, butorphanol, midazolam, propofol and isoflurane in the majority of cases. In two cases where intravenous access was difficult due to severe behavioural resistance or aggression, medetomidine, butorphanol and isoflurane were used. Analgesia was provided primarily with a combination of a non‐steroidal anti‐inflammatory drug and buprenorphine, with local infiltration of lidocaine or bupivacaine added as required. Following wound closure, blood and exudate were carefully cleaned to minimise wound burden, and the surgical site was disinfected with povidone‐iodine with care to avoid contamination of the ear canal. A gauze dressing was applied and the wound was protected with a self‐adhesive elastic bandage, and an Elizabethan collar was fitted.

### Postoperative management

The bandage was removed at approximately 3 to 7 days postoperatively, and suture removal was performed at approximately 12 to 14 days postoperatively. In the latter half of the study period, suture removal was performed under medetomidine sedation in the majority of cases to minimise pain and prevent inadvertent wound trauma during the procedure. Owners were instructed to continue Elizabethan collar use for at least 3 weeks postoperatively to prevent self‐trauma to the surgical site. When removing the collar, owners were instructed to do so gradually under supervision, carefully confirming the absence of self‐trauma, and were repeatedly advised of the high risk of serious consequences resulting from self‐trauma. Owners were encouraged to present for a follow‐up visit at approximately 1 month postoperatively; if no significant problems were identified with the clinical signs or surgical site at that visit, the consultation was concluded and owners were instructed to return if any ear‐related problems arose. In cases with residual clinical signs, topical application of medication to the pinna and horizontal ear canal, ear cleaning or systemic medication was continued as required for the ongoing management of chronic otitis externa.

### Signalment and clinical information

The following information was investigated from medical records: breed, sex, changes in the annual frequency of ear cleaning before and after surgery, the interval from the initial diagnosis of otitis externa to surgery, age at surgery and postoperative follow‐up duration. The annual ear‐cleaning frequency was calculated by referring to the dates of ear cleaning recorded in the medical records and annualising the frequency based on the respective pre‐ and postoperative follow‐up periods.

### Suppurative otitis externa cases

Medical records were reviewed for all cases presenting with purulent discharge from the ear canal or pinna prior to LECR. Items investigated included the duration and character of the purulent discharge, the implementation and results of bacterial culture and sensitivity testing, the circumstances leading to the decision to perform LECR and the details of medical management administered prior to surgery, and the postoperative clinical course.

### Outcome assessment

Clinical outcomes were evaluated based on the criteria described by Sylvestre (1998). To the best of the authors' knowledge, no large‐scale study specifically evaluating the long‐term clinical outcomes of LECR has been published since Sylvestre (1998). In light of this literature gap, Sylvestre (1998) was used as the most methodologically comparable reference available at the present time. It is recognised, however, that direct comparison between studies conducted in different eras, facilities and patient populations has inherent limitations, and this must be considered carefully in the interpretation of results. Outcomes were classified into four categories: Excellent, Good, Fair and Poor. Excellent was defined as no more than two episodes of otitis externa following surgery with no requirement for regular ear cleaning; Good as occasional flare‐ups occurring no more than twice per year that responded readily to topical medical management; Fair as requiring regular ear cleaning to prevent recurrence of otitis and experiencing an average of three or more episodes per year; and Poor as progression to an end‐stage ear with an occluded canal during the follow‐up period or the ear remaining unmanageable.

### Assessment of postoperative anaesthetic procedures and additional surgery

Cases requiring procedures under anaesthesia or sedation related to otitis externa following LECR were investigated, and whether additional surgery had been performed on the LECR‐treated ear was examined. Surgical procedures or interventions considered clearly unrelated to otitis externa or LECR, such as ovariohysterectomy or laparotomy, were excluded from the investigation. Suture removal performed under sedation for the purpose of pain relief and prevention of inadvertent wound trauma was similarly excluded from the investigation.

### Assessment of surgical complications

The following were defined as serious complications and their occurrence was investigated: intraoperative death, periauricular abscess, severe intraoperative haemorrhage, head tilt, nystagmus, persistent discharge from the incision site, wound infection, wound dehiscence, vestibular dysfunction, draining tract formation, deep abscess, facial nerve paralysis, Horner syndrome, neurological deficits and euthanasia. The occurrence of minor complications was similarly investigated.

### Owner assessment

Owner assessments were conducted *via* in‐person interview or telephone at or near the final follow‐up visit at least 12 months postoperatively. Telephone interviews were conducted only for cases in which the dog had already died at the time of data collection. A standardised questionnaire comprising nine items was used. For the first item, owners were asked to quantify the degree of pruritus at the time of assessment as a percentage, with preoperative pruritus defined as 100%. For items two through nine, owners were asked to respond using a five‐point Likert scale. Specifically, owners were asked to assess: the change in pruritus and clinical signs attributable to chronic otitis externa compared with the preoperative period; their impression of discomfort during the period from surgery to suture removal; satisfaction with the postoperative appearance of the surgical site; perceived value relative to the cost of treatment; willingness to recommend LECR to owners of other dogs in a similar situation; overall satisfaction with the procedure, taking into account cost, efficacy and appearance; willingness to choose the same procedure again if placed in the same situation; and whether they felt surgery should have been performed at an earlier stage. Owners were also invited to provide optional free‐text comments. In one case, the interview was discontinued due to the emotional distress experienced by the owner during the assessment, and in one case contact with the owner was lost after the final visit; consequently, the number of respondents for some items was lower than the total number of cases.

### Preoperative informed consent

Prior to LECR, owners were informed that the objectives of surgery included reducing the long‐term management and financial burden associated with chronic otitis externa, and avoiding TECA/LBO, which carries a greater degree of invasiveness and results in hearing loss. Owners were also informed that while a reduction in clinical signs could be anticipated, complete cure of chronic otitis externa could not be guaranteed, and that ear canal stenosis may progress irreversibly without continuation of appropriate medical management. Surgery was performed when the owner was judged to have understood and consented to these explanations. This study was a retrospective study, and all clinical procedures and surgical interventions described were performed as part of routine clinical practice. No additional interventions or procedures were undertaken specifically for the purposes of this study. Prior to treatment, including LECR, written informed consent was obtained from all owners, stating that personal information would not be disclosed and that clinical data and images related to treatment might be used for academic and research purposes. This study was conducted with due consideration for animal welfare and owner interests and in accordance with generally accepted ethical principles for clinical research.

## RESULTS

### Signalment and clinical information

A total of 40 dogs (58 ears) were included in the analysis. Breeds were diverse; Shiba Inu and mixed breeds each accounted for 11 ears (19.0%), followed by Toy Poodles with 9 ears (15.5%). There were 26 males and 14 females. Patient demographics and clinical characteristics are summarised in Table 1. The median age at surgery was 2715.5 days (interquartile range (IQR) 1533.5 to 3488.25), the median interval from the initial diagnosis of otitis externa to surgery was 645.5 days (IQR 131.75 to 1504), and the median postoperative follow‐up duration was 952 days (IQR 589.5 to 1617). At the time of surgery, no clinical or imaging findings consistent with end‐stage ear canal disease were identified in any of the included ears.

**Table 1:** Patient demographics and clinical characteristics of 40 dogs (58 ears) undergoing lateral ear canal resection for chronic otitis externa between May 2016 and March 2025

Variable	Value
Number of dogs	40
Number of ears	58
Median age at surgery, days (IQR)	2716 (1534 to 3488)
Sex (male/female)	26/14
Side (right/left/bilateral)	13/8/19
Median postoperative follow‐up duration, days (IQR)	952 (589.5 to 1617)

Breed distribution among 58 ears: Shiba Inu 11 (19.0%), mixed breeds 11 (19.0%), toy poodles 9 (15.5%), miniature dachshunds 5 (8.6%), shih‐tzus 5 (8.6%), miniature schnauzers 5 (8.6%), Cavalier King Charles spaniels 4 (6.9%), French bulldogs 2 (3.4%), Yorkshire terriers 2 (3.4%), Golden retrievers 2 (3.4%), German shepherds 1 (1.7%), beagles 1 (1.7%) and miniature pinschers 1 (1.7%). Percentages are based on the total number of ears (*n* = 58)

### Suppurative otitis externa cases

Of all cases, six dogs presented with purulent discharge from the ear canal or pinna requiring treatment. Two of these cases were transient and resolved with simple medical management. The remaining four cases are described in detail below. One dog was referred with a chief complaint of longstanding suppurative otitis externa and concurrent head tilt. Following discussion with the owner, a decision was made to proceed with LECR to improve ventilation and drainage, followed by continuation of medical management. Purulent discharge and otitis externa signs resolved following bilateral LECR, and no signs of chronic otitis externa were identified on physical examination at 178 days postoperatively. The head tilt that had been present preoperatively persisted mildly postoperatively but showed clear improvement. Computed tomography to exclude otitis media was not performed due to financial constraints. In one case, otitis media was excluded by computed tomography and a diagnosis of suppurative otitis externa was made. Medical management including bacterial culture and sensitivity testing was proposed, but LECR was prioritised at the owner's request. Two ear cleanings were performed within 2 weeks of suture removal, and no signs of otitis externa have been observed for more than 895 days following suture removal. In one case, bacterial culture and sensitivity testing were proposed due to persistent purulent discharge, but LECR was prioritised at the owner's request. Four ear cleanings were performed within 40 days of suture removal, after which the discharge resolved. This dog died of bronchopneumonia at 964 days postoperatively, and the cause of death was considered unlikely to be related to LECR. In one case, intensive ear cleaning and multiple courses of antimicrobial therapy based on culture and sensitivity testing were administered during outpatient and inpatient management, but no improvement was achieved over a period of more than 3 months. Pseudomonas aeruginosa was isolated on bacterial culture and demonstrated multidrug resistance. The dog also failed to respond to a novel protein diet and antifungal therapy. One ear cleaning was performed 7 days after suture removal, after which the purulent discharge resolved, and no signs of otitis externa have been observed at 553 days postoperatively. Computed tomography to exclude otitis media was not performed due to financial constraints.

### Outcome assessment

Postoperative outcomes were classified as Excellent in 28 ears (48.3%), Good in 12 ears (20.7%), Fair in 18 ears (31.0%) and Poor in 0 ears, resulting in an Acceptable rate (Excellent and Good combined) of 69.0%. No cases progressed to end‐stage ear canal disease or required conversion to TECA/LBO during the follow‐up period. The median annual ear‐cleaning frequency decreased from 6.74 (IQR 2.06 to 14.30) before surgery to 1.58 (IQR 0.42 to 3.77) after surgery. A summary of clinical outcomes is presented in Table 2. Computed tomography images obtained at the time of surgery in the case shown in Figs 1 and 2 are provided as Fig 3.

**Table 2:** Summary of postoperative clinical outcomes and perioperative data for 40 dogs (58 ears) undergoing lateral ear canal resection, classified according to the criteria of Sylvestre (1998)

Variable	Value
*Surgical outcome classification*	
Excellent	28 ears (48.3%)
Good	12 ears (20.7%)
Fair	18 ears (31.0%)
Poor	0 ears (0%)
Acceptable (Excellent + Good)	40 ears (69.0%)
*Annual ear‐cleaning frequency*	
Preoperative, median (IQR)	6.74 (2.06 to 14.30)
Postoperative, median (IQR)	1.58 (0.42 to 3.77)
Postoperative pruritus score, median % (IQR)	20 (10 to 35)
Interval from initial diagnosis to surgery, median days (IQR)	645.5 (131.75 to 1504)
Age at surgery, median days (IQR)	2715.5 (1533.5 to 3488.25)
Postoperative follow‐up duration, median days (IQR)	952 (589.5 to 1617)

**FIG 1:**
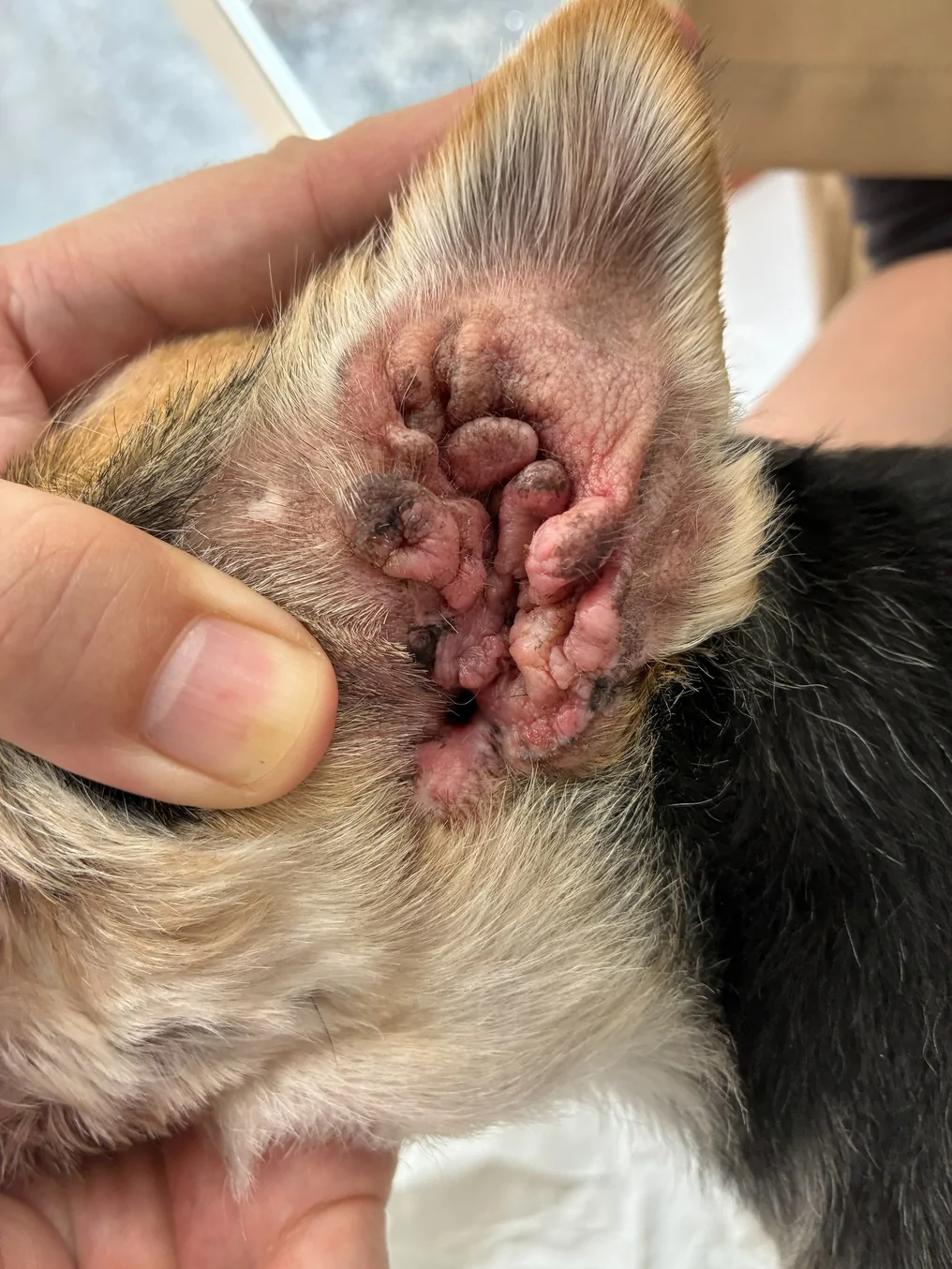
External appearance of the left ear 448 days after left‐sided LECR. Proliferative pinnal lesions that developed and accumulated over the postoperative course are observed. The postoperative outcome was classified as Fair. It should be noted that the contralateral right ear underwent simultaneous TECA/LBO at the time of left‐sided LECR, with complete resolution of chronic otitis externa signs.

**FIG 2:**
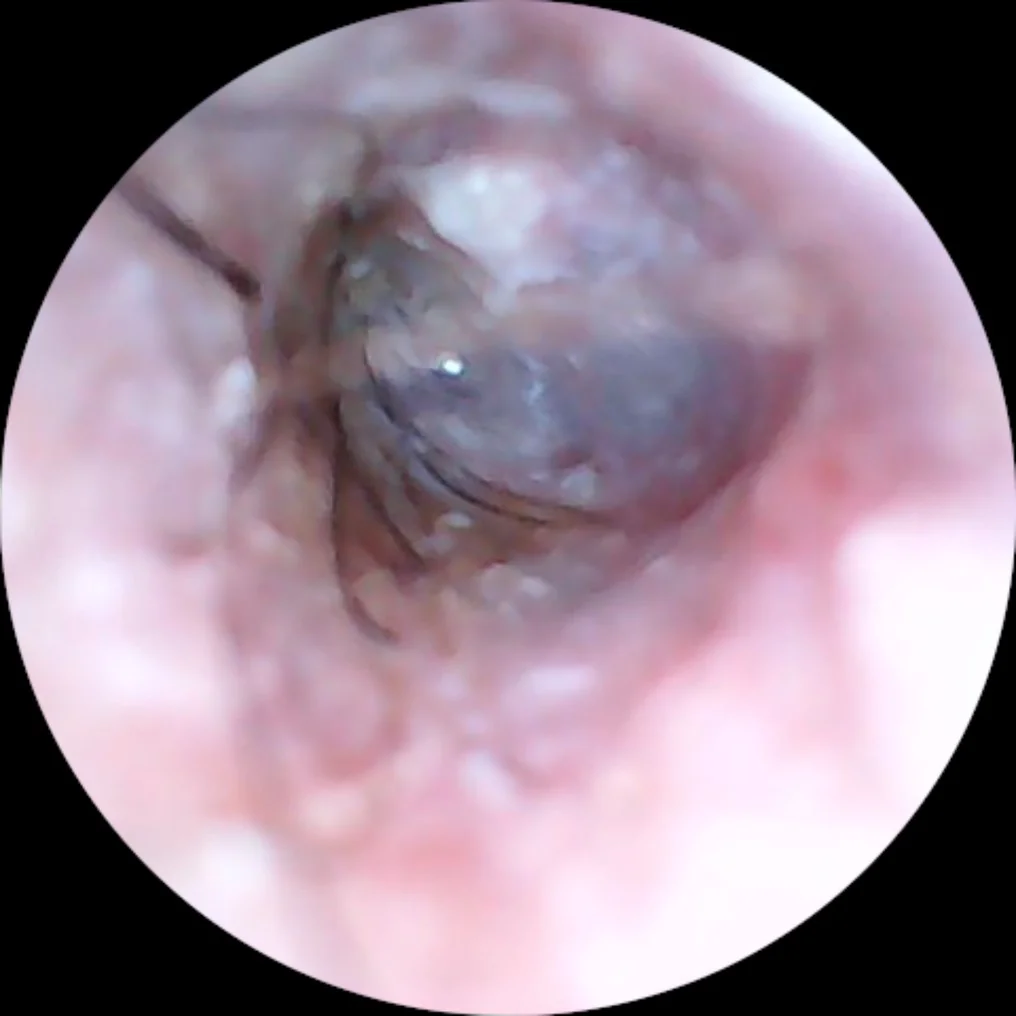
Otoscopic image of the case shown in Fig 1, obtained at the final follow‐up visit (448 days postoperatively). The patency of the horizontal ear canal is well maintained, with no marked stenosis identified. Mild inflammatory changes are observed along the canal wall in the region of the tympanic membrane; however, adequate luminal space is preserved throughout the horizontal ear canal.

**FIG 3:**
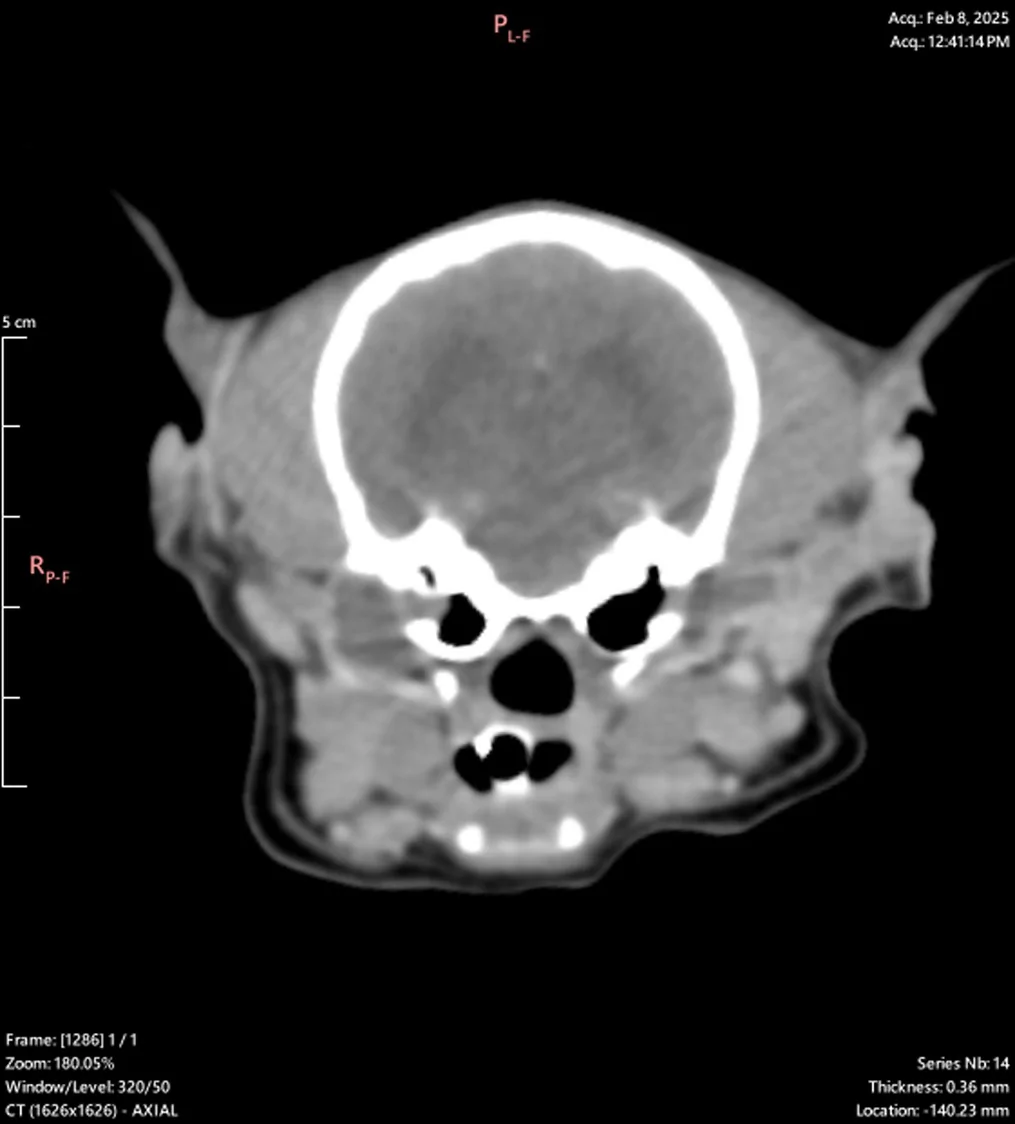
This image represents a computed tomography scan obtained at the time of surgery in the case shown in Figs 1 and 2. Bilateral ear canal stenosis was identified at the initial presentation. As the dog resided at a considerable distance from the hospital and frequent visits were not feasible, a decision was made in consultation with the owner to perform TECA/LBO on the right ear and LECR on the left ear. Preoperative computed tomography revealed fluid accumulation in the region of the horizontal ear canal of the right ear. The postoperative outcome of the LECR‐treated left ear was classified as Fair.

### Postoperative anaesthetic procedures and additional surgery

Two dogs (3 ears) required procedures under anaesthesia or sedation related to otitis externa following LECR. The first was an entire male mixed‐breed dog with an age at surgery of 1010 days, an interval from initial diagnosis of otitis externa to surgery of 13 days, and a postoperative follow‐up duration of 939 days. Ear cleaning was attempted at the initial visit, but severe aggression made it difficult to ensure the safety of the owner and hospital staff; bilateral LECR was therefore performed following discussion with the owner. Strong pruritus persisted postoperatively, and medical management with oral oclacitinib, ilunocitinib and itraconazole, subcutaneous prednisolone injection, a novel protein diet, topical preparation containing fluocinonide and topical ketoconazole was attempted, but improvement was limited. The owner‐reported postoperative pruritus score was 75%. A total of 24 ear cleanings were performed after suture removal, requiring three sessions of sedation with medetomidine and butorphanol and two sessions of general anaesthesia with medetomidine, butorphanol and isoflurane. The second dog was an entire male Shiba Inu that underwent LECR of the left ear, with an age at surgery of 3075 days, an interval from initial diagnosis to surgery of 152 days and a postoperative follow‐up duration of 872 days. This dog was highly aggressive, and the first ear cleaning as the initial otitis externa consultation was performed under general anaesthesia at the time of Meibomian gland excision. Only one ear cleaning was performed prior to LECR. At the next otitis externa visit, otoscopic examination under sedation revealed ear canal stenosis, and LECR was performed following discussion with the owner. Three ear cleanings were performed postoperatively, requiring two sessions of sedation with medetomidine and butorphanol due to aggression. This dog underwent LECR of the right ear 593 days after left‐sided LECR; however, as the postoperative follow‐up period for the right ear was less than 12 months, the right ear was excluded from this study.

One dog (1 ear) required reoperation. This was an entire female French Bulldog that underwent LECR of the right ear, with an age at surgery of 754 days, an interval from initial diagnosis to surgery of 522 days and a postoperative follow‐up duration of 1020 days. The preoperative annual ear‐cleaning frequency was 16 times, which decreased to five times following LECR. Purulent discharge and otitis externa signs recurred at 864 days after LECR, and a proliferative polypoid inflammatory lesion was removed from the right horizontal ear canal under general anaesthesia at 911 days postoperatively. The lesion was solitary and pedunculated, and based on its size, an origin from the tympanic bulla, auditory tube or pharynx was considered likely. Clinical signs resolved following reoperation. At the final follow‐up visit 105 days after reoperation, otoscopic examination revealed a polypoid space‐occupying lesion in the region of the tympanic membrane; however, the opening of the horizontal ear canal was maintained and no clinical signs were present, and the case was managed conservatively. The otoscopic image at the final follow‐up visit is shown in Fig 4, a photograph of the removed polyp is shown in Fig 5, and the external appearance at the final follow‐up visit is shown in Fig 6.

**FIG 4:**
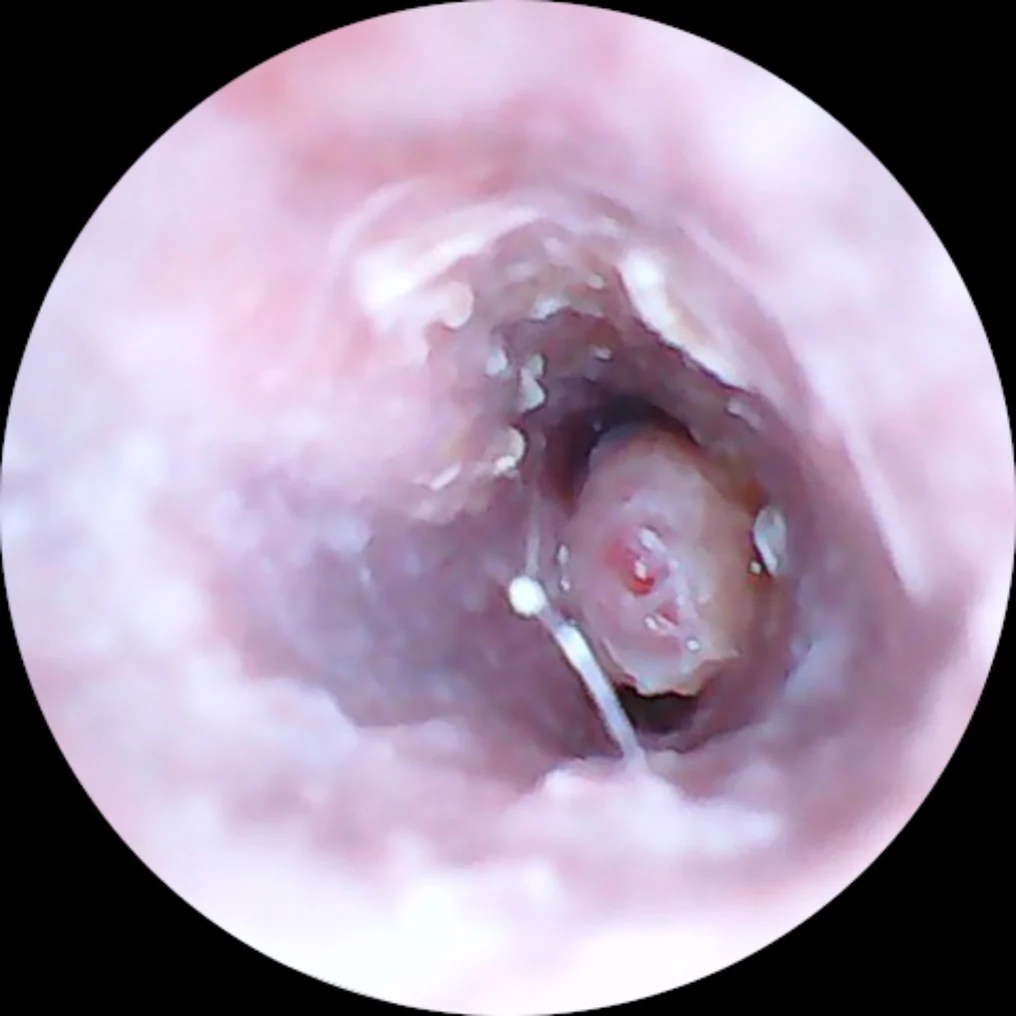
Otoscopic image obtained 1020 days after LECR (105 days after revision surgery) in an ear that required reoperation for surgical excision of a proliferative polypoid lesion. A polypoid lesion was observed in the region of the tympanic membrane; however, the opening of the horizontal ear canal remained patent. No clinical signs of otitis externa were present at this time, and the case was managed with continued observation.

**FIG 5:**
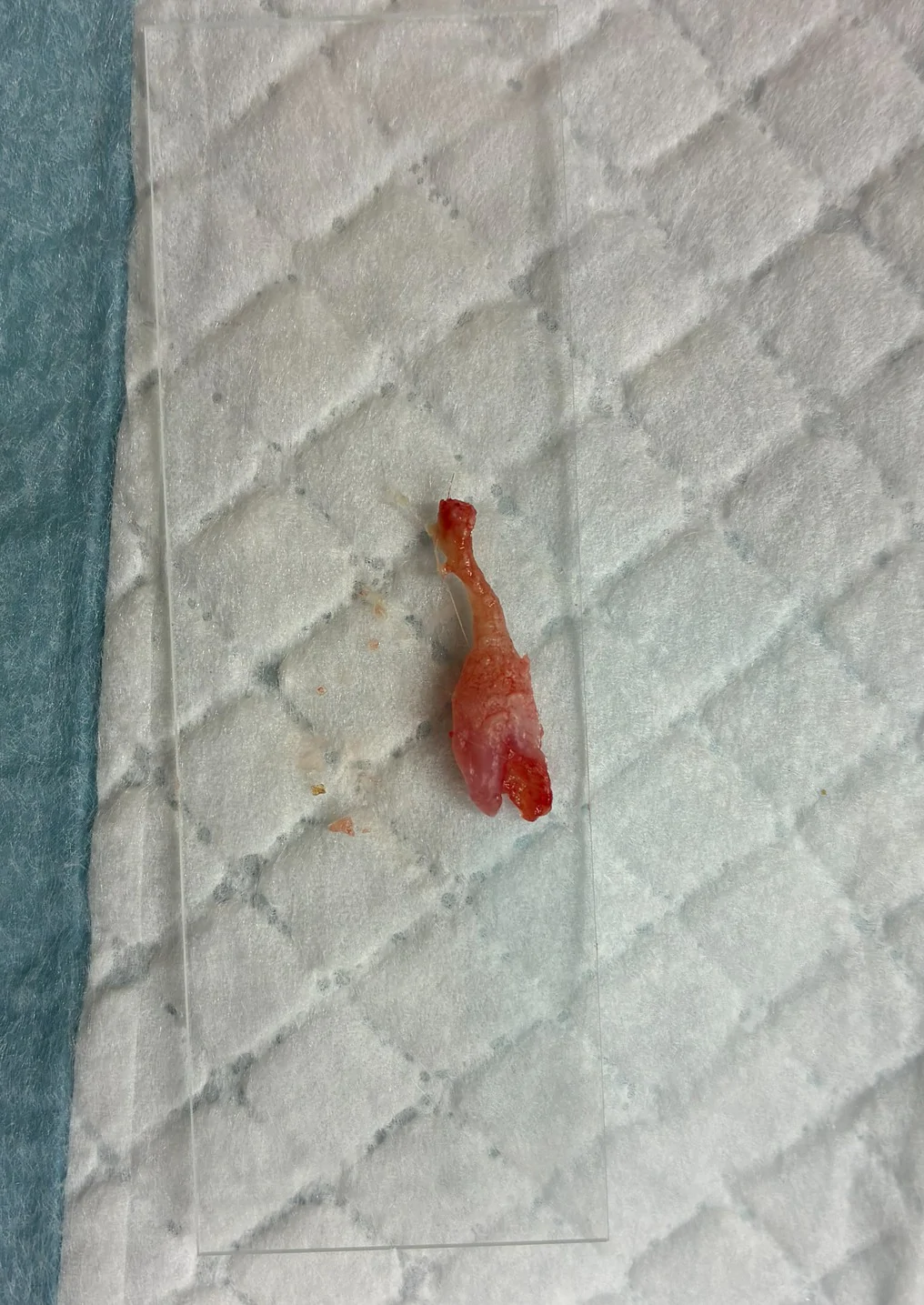
Photograph of a proliferative polypoid lesion excised from the horizontal ear canal in a revision case (Fig 4). The lesion was pedunculated and solitary. Based on its size and morphology, it was considered most likely to have originated from the tympanic bulla, auditory tube or nasopharynx.

**FIG 6:**
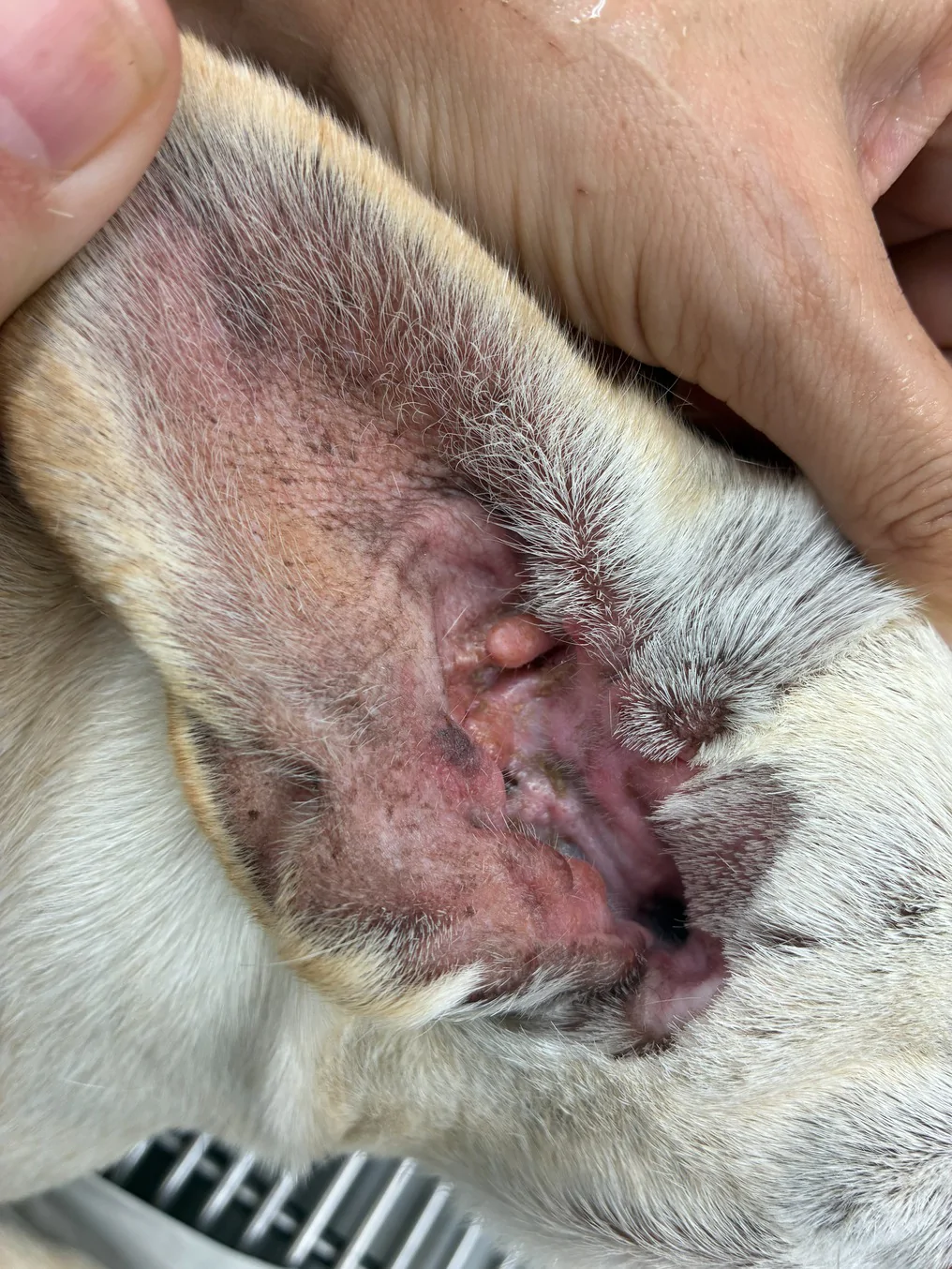
External appearance at 1020 days after surgery, as shown in Fig 4. Although inflammatory changes and hyperpigmentation of the pinna were observed, the clinical course was favourable and the postoperative outcome was classified as Excellent.

### Assessment of surgical complications

No serious complications were observed in any case, including intraoperative death, severe intraoperative haemorrhage, head tilt, nystagmus, persistent discharge from the incision site, severe wound infection, wound dehiscence, vestibular dysfunction, draining tract formation, deep abscess, facial nerve paralysis, Horner syndrome, neurological deficits or euthanasia. One case that presented with preoperative head tilt showed improvement postoperatively; this was considered a preoperative finding and was not classified as a postoperative complication. Minor complications included mild discharge in three cases between 5 and 32 days postoperatively, minor bleeding due to self‐trauma in three cases between 7 and 28 days postoperatively, retained suture material in two cases, and a small amount of purulent material identified at bandage removal in one case. All of these resolved spontaneously or with short‐term treatment. In one case where medetomidine was used due to difficulty obtaining intravenous access because of behavioural factors, transient bradycardia (heart rate 40 BPM) was observed intraoperatively; this resolved spontaneously without affecting the postoperative course and was considered to be within the known pharmacological effects of medetomidine. Although a direct causal relationship with the surgery could not be established, a subcutaneous abscess was identified on the surgical side in one case at 473 days after LECR, which resolved completely with treatment without anaesthesia and a 10‐day course of amoxicillin. In one additional case, an aural haematoma developed at 876 days after LECR and resolved with standard treatment within 49 days.

### Owner assessment

The median postoperative pruritus score, expressed as a percentage of the preoperative pruritus level defined as 100%, was 20% (IQR 10 to 35), with a mean ± SD of 27.41% ± 22.27%. Likert scale assessment results are presented in Table 3. Mean scores exceeded 3.89 across all items and exceeded 4.14 for all items except the impression of perioperative discomfort (3.89 ± 0.87). Particularly high scores were obtained for satisfaction with postoperative appearance (4.81 ± 0.44), overall satisfaction with the procedure (4.77 ± 0.50), and willingness to choose the same procedure again (4.75 ± 0.44). The mean score for preference for earlier surgical intervention was 4.14 ± 0.90, indicating a tendency toward preferring earlier surgery. In the free‐text responses, positive comments regarding reduction in postoperative pruritus and decreased burden of veterinary visits and ear cleaning were predominant, and many owners expressed a preference for earlier surgical intervention or noted that the procedure had been less burdensome than anticipated prior to surgery. These free‐text responses were broadly consistent with the quantitative Likert scale assessment results.

**Table 3:** Owner assessment scores using a five‐point Likert scale, obtained at the final follow‐up visit at least 12 months postoperatively

Assessment item	*n*	Mean ± SD	5	4	3	2	1
Change in pruritus and clinical signs	57	4.56 ± 0.66	37	15	5	0	0
Discomfort during the perioperative period	56	3.89 ± 0.87	12	32	6	6	0
Satisfaction with postoperative appearance	57	4.81 ± 0.44	47	9	1	0	0
Perceived value relative to cost	58	4.38 ± 0.79	31	20	5	2	0
Willingness to recommend LECR to other owners	56	4.34 ± 0.67	25	25	6	0	0
Overall satisfaction with the procedure	56	4.77 ± 0.50	45	9	2	0	0
Willingness to choose the same procedure again	56	4.75 ± 0.44	42	14	0	0	0
Preference for earlier surgical intervention	56	4.14 ± 0.90	25	16	13	2	0

For all items, 5 represents the most favourable response and 1 represents the least favourable response. Change in pruritus and clinical signs: 5 = much improved, 1 = much worse. Discomfort during the perioperative period: 5 = no discomfort, 1 = completely unacceptable. Satisfaction with postoperative appearance: 5 = very satisfied, 1 = very dissatisfied. Perceived value relative to cost: 5 = very satisfied, 1 = very dissatisfied. Willingness to recommend LECR to other owners: 5 = strongly recommend, 1 = strongly not recommend. Overall satisfaction with the procedure: 5 = very satisfied, 1 = very dissatisfied. Willingness to choose the same procedure again: 5 = definitely yes, 1 = definitely no. Preference for earlier surgical intervention: 5 = strongly agree that surgery should have been performed earlier, 1 = strongly agree that surgery should have been delayed

The number of respondents (*n*) was less than the total number of cases for some items because one owner became emotionally distressed during the interview and requested to stop, and one owner declined to respond despite having a clinical record.

Fig 7 shows the external appearance at the final follow‐up visit of a representative case in which proliferative pinnal lesions developed and accumulated following LECR, suggesting that the case would likely have progressed to a condition requiring TECA/LBO had appropriate surgical intervention not been performed.

**FIG 7:**
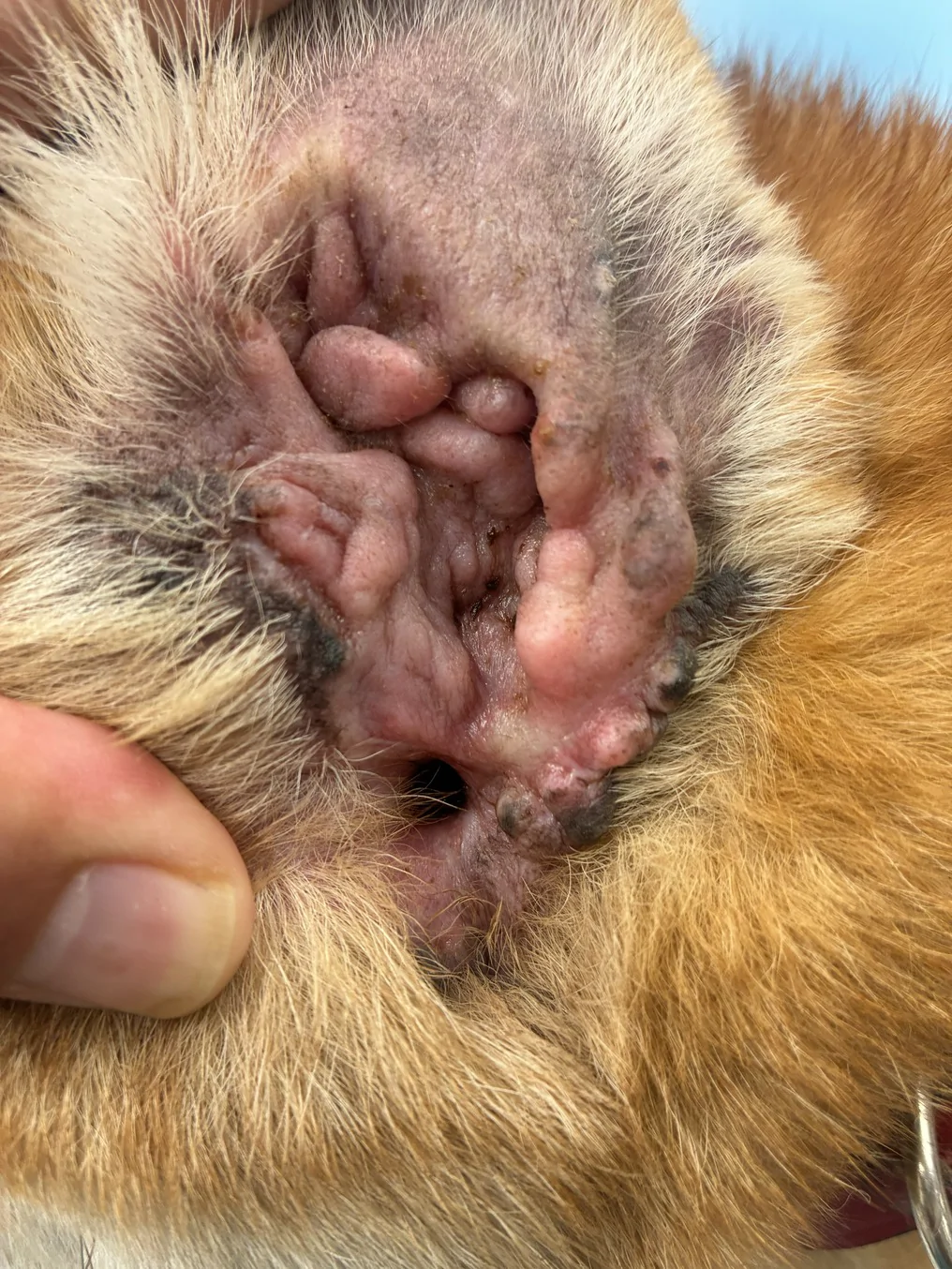
This image was obtained 1892 days after surgery and does not represent the preoperative condition. The proliferative changes observed here developed progressively following the procedure. Although the auricular lesions advanced over time, the horizontal ear canal remained patent, suggesting that LECR may have contributed to preventing progression to end‐stage ear canal obstruction. The postoperative outcome was classified as Good.

Fig 1 shows the external appearance of a representative case classified as Fair at 12 to 24 months (448 days) postoperatively, and Fig 2 shows the otoscopic image of the same case at the final follow‐up visit.

Fig 8 shows the external appearance of a separate representative case classified as Fair at 897 days postoperatively, and Fig 9 shows the otoscopic image of the horizontal ear canal and tympanic membrane of the same case.

**FIG 8:**
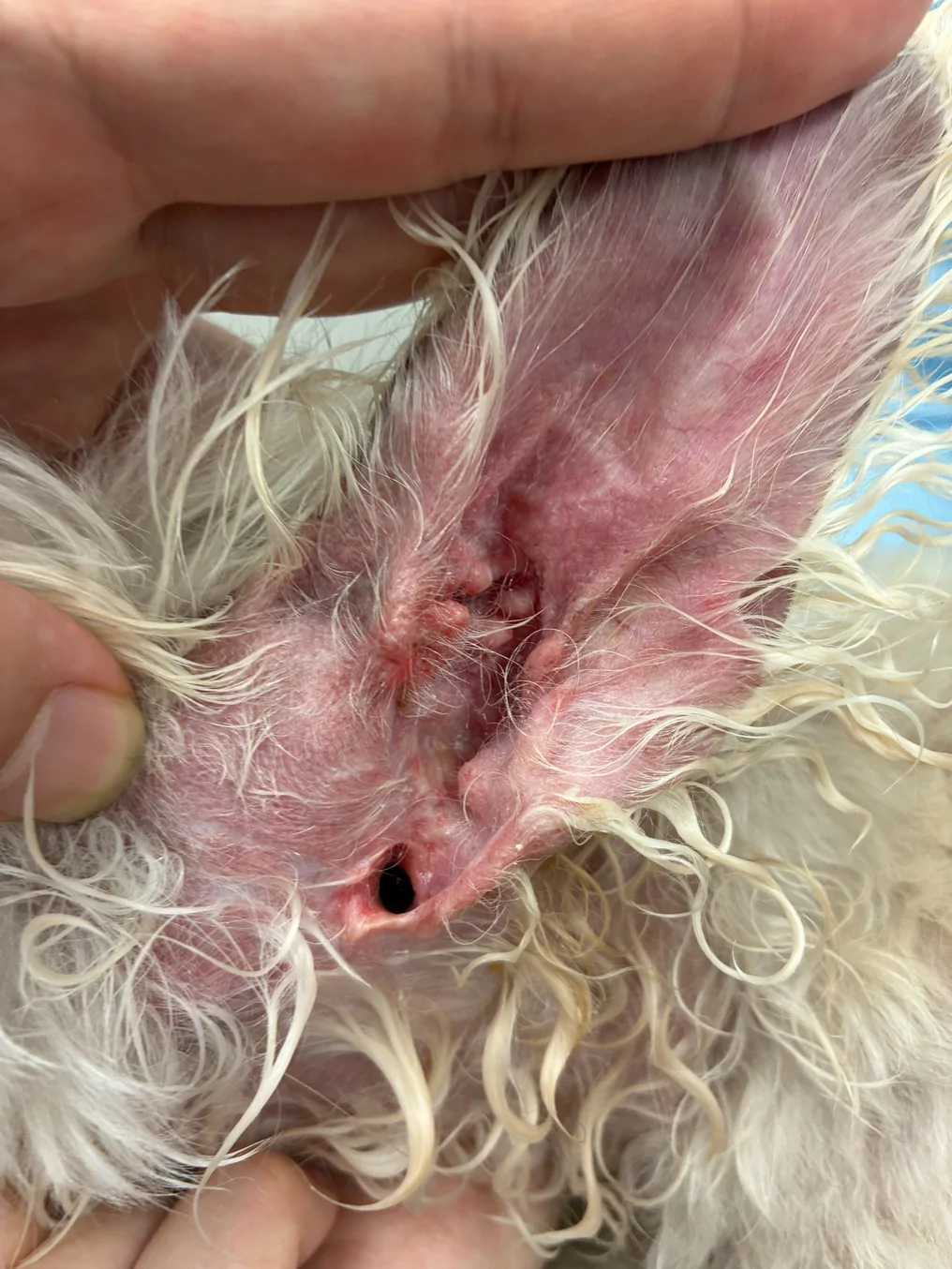
External appearance of a case 897 days after LECR. The patient continued to require regular ear cleaning postoperatively, and inflammatory changes of the pinna were observed. The opening of the horizontal ear canal remained well patent. The postoperative outcome was classified as Fair.

**FIG 9:**
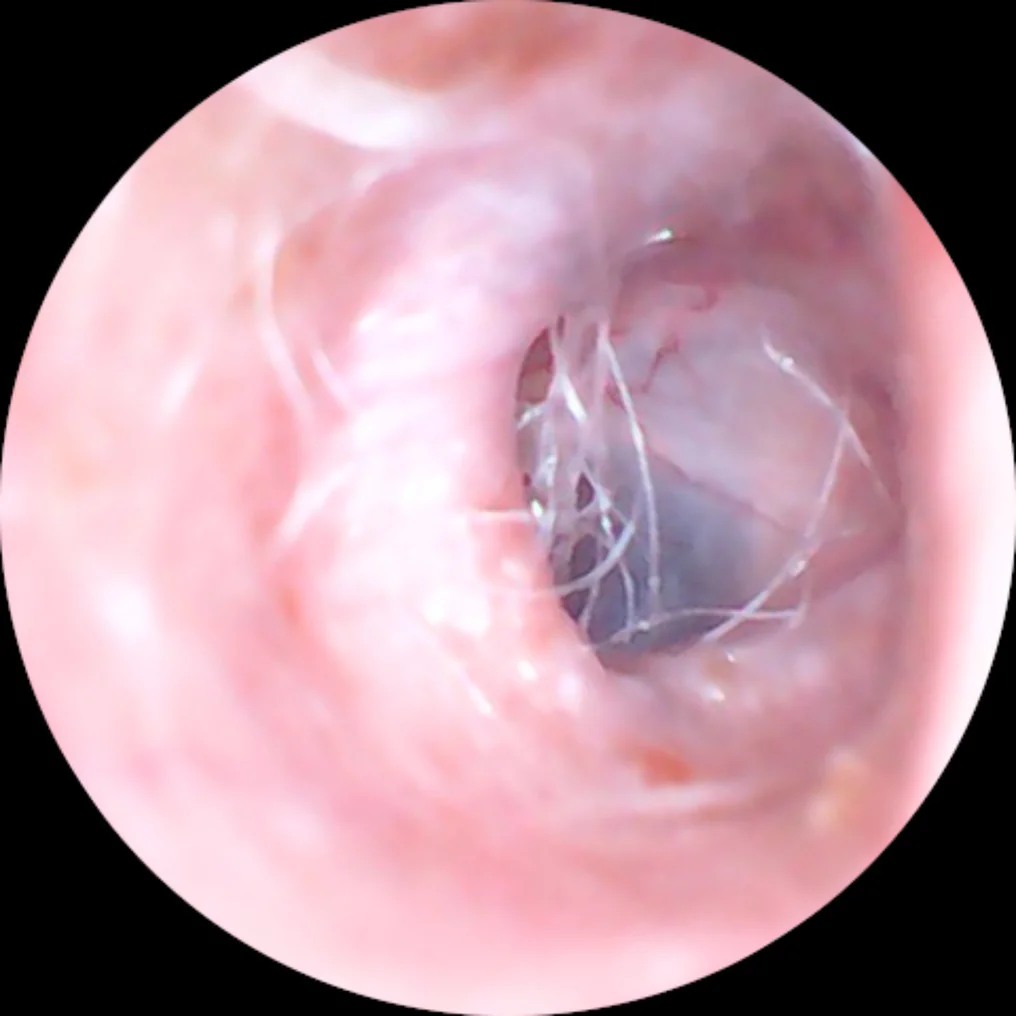
Otoscopic findings of the horizontal ear canal and tympanic membrane 897 days after surgery, as shown in Fig 8. No stenosis of the horizontal ear canal was observed, and no marked inflammatory changes were present. The tympanic membrane appeared normal.

Fig 10 shows the external appearance of a representative case classified as Excellent at 12 to 24 months (618 days) postoperatively.

**Fig 10:**
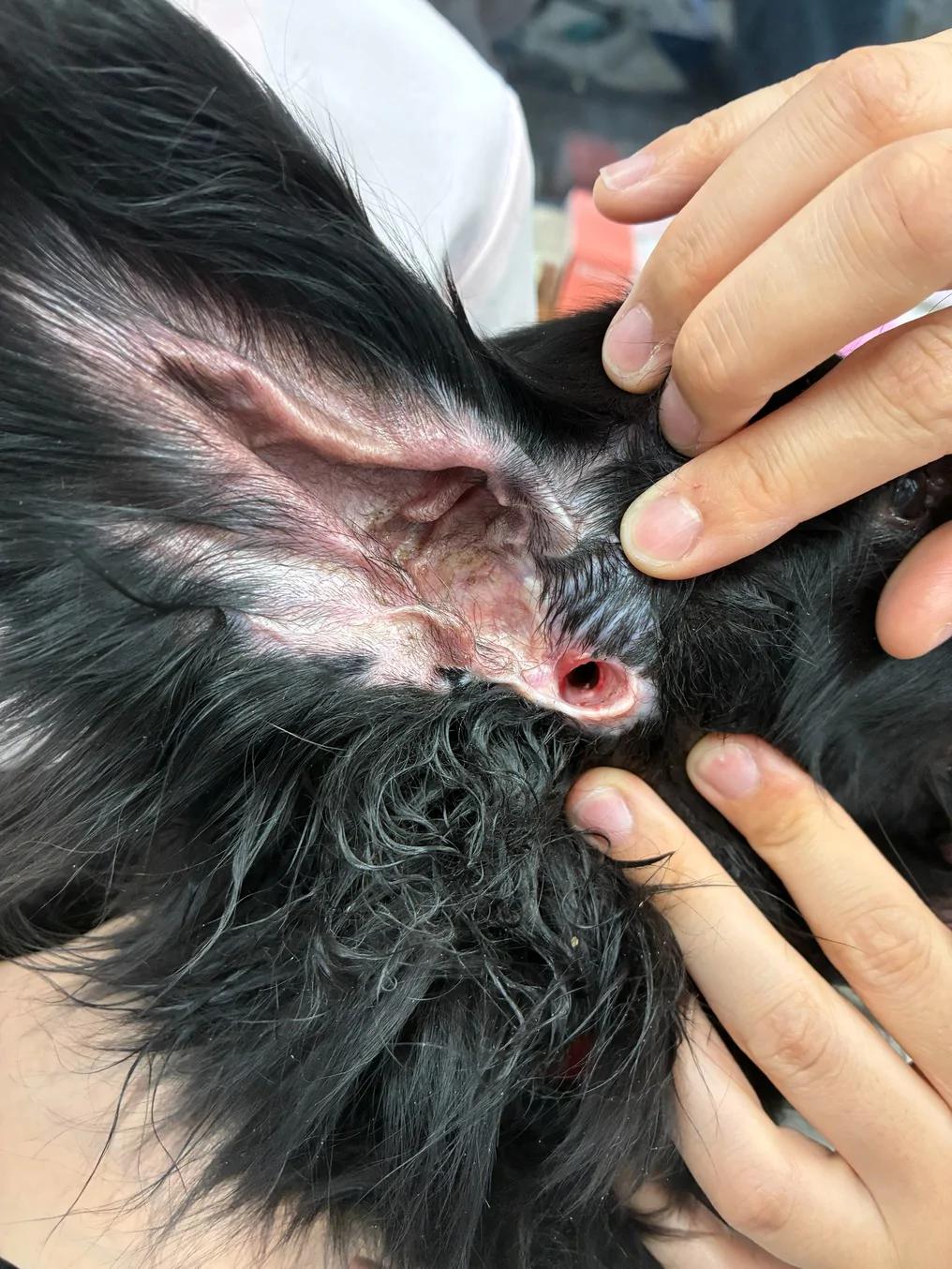
External appearance 618 days after LECR. The postoperative outcome was classified as Excellent. Mild inflammatory changes of the pinna are observed; however, the opening of the horizontal ear canal is well maintained.

## DISCUSSION

To the best of the authors' knowledge, large‐scale reports specifically evaluating the long‐term clinical outcomes of LECR are limited to a small number of publications, including Tufvesson (1955), Gregory and Vasseur (1983) and Sylvestre (1998), and no report of comparable scale has been identified since 2000. Gregory and Vasseur (1983) reported 26 dogs, and Doyle et al. (2004) reported only 9 LECR cases as a subgroup within a larger surgical series. In the context of this literature gap, the conventional view that LECR has limited utility because TECA/LBO is ultimately required cannot be considered to be based on sufficiently large‐scale, long‐term follow‐up data (Doyle et al., 2004; Gregory & Vasseur, 1983; Sylvestre, 1998; Tufvesson, 1955). The present study includes 58 ears, a case series of comparable scale to Sylvestre (1998), and represents, to the best of the authors' knowledge, the largest LECR outcome report published since 2000. The study also contributes to the re‐evaluation of the clinical significance of LECR from a contemporary perspective, in that LECR was performed under medical management informed by current knowledge of chronic otitis externa, including the PSPP framework. It should be noted that this study is a retrospective descriptive study, and the findings should be interpreted as hypothesis‐generating.

An important feature of the present study is that LECR was not applied selectively based on predefined staging criteria, but was broadly incorporated into routine clinical practice whenever chronic otitis externa was considered difficult to manage with medical treatment alone. Furthermore, the operating surgeon held no specialist qualification in either surgery or internal medicine, a video otoscope was not available, and computed tomography was inaccessible for 7 of the 9 years comprising the study period. Despite this pragmatic, non‐selective approach and limited equipment environment, no cases progressed to end‐stage ear canal disease or required conversion to TECA/LBO during the follow‐up period, in contrast to the 17.6% rate of such progression reported by Sylvestre (1998). No serious postoperative complications were observed, and the median annual ear‐cleaning frequency decreased from 6.74 (IQR 2.06 to 14.30) before surgery to 1.58 (IQR 0.42 to 3.77) after surgery, indicating a substantial reduction in long‐term management burden.

With respect to postoperative anaesthetic procedures, two dogs required general anaesthesia or sedation for routine ear cleaning due to severe behavioural resistance or aggression; specifically, one dog required general anaesthesia and the other required sedation. These cases were attributed to patient‐specific behavioural characteristics rather than problems with the management of chronic otitis externa and were classified as Fair and Good, respectively, according to the Sylvestre criteria. With the exception of these cases, no anaesthetic or sedation procedures were required for the purpose of managing chronic otitis externa in any case. One dog required surgical removal of a proliferative polypoid lesion arising from a region deep to the tympanic membrane on the LECR‐treated side. As shown in Figs 4 and 6, the opening of the horizontal ear canal and its lumen were maintained at the time of reoperation. This dog also exhibited generalised pruritus considered to be attributable to an underlying allergic predisposition, and it is considered more likely that the polypoid lesion originated from a region deep to the tympanic bulla through a mechanism distinct from the direct progression of chronic otitis externa. Accordingly, the fact that reoperation was required was not considered to diminish the effectiveness of LECR in managing chronic otitis externa in this case. In addition, no case in this study required referral to a facility equipped with a video otoscope, and long‐term disease control was maintained throughout the follow‐up period without recourse to TECA/LBO.

These findings support the concept that LECR may contribute to suppressing progression of recurrent otitis externa while maintaining long‐term manageability. The results further suggest that LECR can be performed in a primary care setting without access to advanced specialist facilities or equipment, provided appropriate case selection is applied, and that the procedure may contribute to avoiding progression to TECA/LBO in similar clinical contexts. It should be noted that these observations carry no implication that video otoscopy lacks utility; it is self‐evident that numerous cases exist for which a video otoscope or TECA/LBO would be indispensable. Suppurative otitis externa, particularly cases involving *Pseudomonas* spp., is known to frequently demonstrate treatment resistance and can be difficult to manage (Secker et al., 2023). The observation that a proportion of cases with persistent purulent discharge refractory to medical treatment in the present study showed resolution of clinical signs following LECR with relatively straightforward medical management is an interesting finding that warrants further investigation.

The indications for TECA/LBO are well established, being reserved for end‐stage chronic otitis externa in which no other effective treatment option remains—a state of irreversible pathological change. By contrast, precisely defining the indications for LECR is inherently more difficult. Prior to the development of end‐stage disease, the degree of ear canal inflammation, thickening and stenosis varies along a continuous spectrum from case to case, and these changes are not necessarily unidirectional, as partial reversibility may occur. Accurate preoperative prediction of long‐term ear canal progression over the medium to long term remains challenging, and this difficulty is considered one reason why the indications for LECR have not been clearly established historically. It is postulated that this background, in contrast to the well‐defined indications for TECA/LBO, underlies the relative paucity of detailed investigation into the clinical utility of LECR.

Against this background, the findings of the present study may offer one potential clinical reference point. The median annual ear‐cleaning frequency decreased markedly after surgery, owner satisfaction scores were high across all assessed domains, and no cases required conversion to TECA/LBO during the follow‐up period. These results paradoxically suggest that the point at which the preoperative annual ear‐cleaning frequency reaches a certain level may serve as a reference when considering LECR. While it is not possible to conclude that this represents the optimal timing for surgery given the nature of this study, the use of annual ear‐cleaning frequency as one clinical reference criterion when considering LECR may have practical utility. Validation of this finding in future prospective studies is anticipated.

With regard to whether the cases included in the present study truly represented conditions requiring LECR, the authors recognise that no definitive counter‐argument can be made, given the inherent difficulty in defining the optimal timing for LECR described above, in addition to the absence of systematically recorded objective data regarding the degree of preoperative ear canal stenosis, otoscopic findings and histopathological findings. However, for the 12 ears represented by Figs 1 and 7, progressive formation and accumulation of proliferative pinnal lesions were confirmed over the postoperative course, and it is considered likely that these cases would have progressed to a condition requiring TECA/LBO had appropriate surgical intervention not been performed. The fact that the horizontal ear canal opening has been maintained in these cases at the present time is considered one piece of evidence that LECR was effective in suppressing disease progression in at least these cases. It should be noted that these cases are not representative of all cases in the present study, and this observation is limited to those for which imaging findings were available.

TECA/LBO is an effective treatment; however, its postoperative complications are diverse. Recent reports indicate that the overall complication rate ranges from 21% to 82% (Aranda‐Jiménez et al., 2023; Banks et al., 2023), with individual complications including neurological complications such as facial nerve paralysis in 3% to 58%, vestibular disease in 1% to 23% and Horner syndrome in 3% to 67%, intraoperative complications including intraoperative death in 4.2% and severe intraoperative haemorrhage in 5.5% to 15.6%, and infection‐related reoperation required in 11.2% of cases (Banks et al., 2023; Folk et al., 2022; Szewczyk et al., 2025). In contrast, no serious complications were observed in any case in the present study, and minor complications that were identified in a small number of cases resolved spontaneously or with short‐term treatment. No studies directly comparing LECR and TECA/LBO in terms of technical difficulty, financial burden, invasiveness, complication rate and safety profile have been identified within the authors' literature search. However, it is generally considered that LECR may be associated with lower technical difficulty, lower financial burden, lower invasiveness and potentially fewer complications compared with TECA/LBO, and the procedure may be a viable option in primary care settings. Although a direct comparison with TECA/LBO has inherent limitations due to differences in study design and the nature of the patient populations, the safety profile of LECR when performed with appropriate case selection may be more favourable than that of TECA/LBO.

Previous studies and textbook descriptions have suggested that owner satisfaction following LECR for canine chronic otitis externa is not necessarily high (Fossum, 2019) and have emphasised the importance of thorough preoperative counselling regarding the fact that chronic otitis externa is not a surgically curable disease and requires lifelong management. In the present study, repeated explanations were provided regarding the purpose of LECR, the progressive and irreversible nature of horizontal ear canal stenosis in the end stage, the invasiveness and risks associated with TECA/LBO and the necessity of continued long‐term management after surgery. Such preoperative counselling may have contributed to appropriate alignment of treatment expectations and to the high owner satisfaction observed in this study. The finding that many owners felt surgery should have been performed at an earlier stage suggests that there may be a discrepancy between the point at which veterinarians propose surgical intervention and the point at which owners perceive a substantial decline in their dog's wellbeing. It has been reported that in advanced chronic otitis externa, cumulative pathological changes associated with chronic inflammation increase treatment difficulty, complication risk and financial burden (Nuttall, 2023), and presenting LECR as a treatment option at an earlier stage in the disease course may contribute to improved long‐term management outcomes.

In conclusion, LECR performed as part of routine clinical practice before the development of overt end‐stage ear canal disease may suppress progression of chronic otitis externa and support long‐term disease manageability. In contemporary otitis management, LECR warrants re‐evaluation not only as a salvage procedure but also as a treatment option to be considered earlier in the disease course. Future multicentre prospective studies are needed to confirm the generalisability of these findings.

This study has several limitations. First, this was a retrospective analysis conducted at a single institution by a single surgeon, with no formal inclusion criteria, prospective disease staging or control group. Selection bias cannot be completely excluded, and this limits the ability to precisely define the optimal indications or timing for LECR. Second, objective data regarding the degree of preoperative ear canal stenosis, otoscopic findings and histopathological findings were not systematically recorded, preventing detailed assessment of the relationship between disease severity at the time of surgery and postoperative outcome. Third, no structured postoperative monitoring plan was established; instead, assessments were conducted when owners presented with otitis externa‐related complaints. This approach carries the risk of missing subtle clinical signs and inconsistent decision‐making regarding intervention, which may have influenced the assessment of postoperative outcomes. Furthermore, no systematic guidance was provided to owners regarding the recognition of subtle clinical signs, which represents a similar limitation. Fourth, although ear cleaning was followed by otoscopic confirmation of cerumen and discharge removal in all cases, complete visualisation of the tympanic membrane was not achieved in many cases, and systematic assessment of tympanic membrane pathology was therefore not performed. Fifth, owner‐reported assessments were based on subjective questionnaires and may be subject to response bias. Despite these limitations, the consistent observation that no cases progressed to end‐stage disease or required conversion to TECA/LBO during the long follow‐up period (median 952 days) is a clinically meaningful finding suggesting the potential utility of LECR when performed before the development of overt end‐stage changes. Future multicentre prospective studies are needed to confirm the generalisability of these findings.

## Author contributions

**K. Iyori:** Conceptualization; methodology; visualization; supervision. **A. Yabuzoe:** Conceptualization; methodology; formal analysis; supervision. **K. Kiriki:** Conceptualization; formal analysis; investigation; resources; data curation; writing – original draft.

## Conflict of interest

The authors declare no conflicts of interest.

## AI disclosure statement

Artificial intelligence tools were used to assist with English‐language editing and manuscript drafting. Specifically, ChatGPT (OpenAI) and Claude (Anthropic) were used for language refinement. The authors reviewed and approved all final content. No AI tools were used for study design, data analysis, outcome assessment or interpretation of results.

## Supporting information


**Fig S1.** External appearance of a Shiba Inu at 1288 days postoperatively. Erythema and hyperpigmentation are observed on the pinna and surrounding skin. Exudate and ceruminous debris were minimal. Mild narrowing of the horizontal ear canal opening is noted on external examination; however, patency is well maintained. This case, Figures S2–S10, and Figures 1 and 7 — 12 ears in total — exhibited particularly marked proliferative lesions following LECR. None of these images represent the preoperative condition; the proliferative changes observed developed progressively after surgery. Had LECR not been performed, it is considered likely that these cases would have ultimately progressed to a condition requiring TECA/LBO.
**Fig S2.** External appearance of a Shiba Inu at 1773 days postoperatively (a different individual from S1). Erythema and hyperpigmentation are observed on the pinna and surrounding skin. Exudate and ceruminous debris are minimal, and patency of the horizontal ear canal is maintained.
**Fig S3**. External appearance of the right ear of a Toy poodle at 1496 days postoperatively. Proliferative lesions, lichenification and hyperpigmentation are observed on the pinna. In contrast to the pinnal changes, patency of the horizontal ear canal opening is well maintained.
**Fig S4**. External appearance of the left ear of the same individual as S3 at 1496 days postoperatively. As with the contralateral ear, proliferative lesions, lichenification and hyperpigmentation are observed on the pinna, whereas patency of the horizontal ear canal opening is maintained.
**Fig S5**. External appearance of a Shiba Inu at 582 days postoperatively. Despite the relatively short postoperative interval, proliferative lesions, hyperpigmentation and erythema of the pinna, along with narrowing of the horizontal ear canal opening, are already observed. Exudate and ceruminous debris are minimal, the owner did not consider the otitis externa signs to be a significant concern and the overall clinical course has been satisfactorily maintained.
**Fig S6**. External appearance of a mixed‐breed dog at 666 days postoperatively. This case demonstrated persistent and marked pruritus following surgery, requiring routine ear canal cleaning; however, due to pronounced aggression, anaesthesia was frequently necessary for each cleaning procedure. Details of the postoperative course are described in the “Results” section under “Postoperative Anaesthetic Procedures and Additional Surgery.”
**Fig S7**. External appearance of the right ear of a Cavalier King Charles Spaniel at 709 days postoperatively. This case exhibited relatively persistent postoperative pruritus and required frequent clinic visits, particularly during warmer seasons. Erythema is observed; however, patency of the horizontal ear canal is maintained.
**Fig S8**. External appearance of the left ear of the same individual as S7 at 709 days postoperatively. Erythema is observed as in the contralateral ear, and some exudate is also noted; however, patency of the horizontal ear canal is maintained.
**Fig S9**. External appearance of the right ear of a Miniature dachshund at 1072 days postoperatively. Severe proliferative lesions and purulent exudate are observed, with narrowing also evident in the horizontal ear canal. Despite consistently high owner compliance, this case represents one of the least favourable outcomes in the present study, and owner satisfaction was relatively lower compared with other cases. Owner questionnaire responses were as follows: Change in pruritus and clinical signs 3, Discomfort during the perioperative period 5, Satisfaction with postoperative appearance 5, Perceived value relative to cost 4, Willingness to recommend LECR to other owners 3, Overall satisfaction with the procedure 4, Willingness to choose the same procedure again 5, Preference for earlier surgical intervention 2, Postoperative pruritus score 50%. Nevertheless, TECA/LBO has not been required as of 1400 days postoperatively.
**Fig S10**. External appearance of the left ear of the same individual as S9 at 680 days postoperatively. Findings consistent with those observed in the contralateral ear are noted.

## Data Availability

Anonymised clinical data and representative case images supporting the findings of this study are available as supplementary data. All data and images were appropriately anonymised to prevent identification of individuals and are disclosed with the informed consent of the owners.
